# Usage of Multilingual Mobile Translation Applications in Clinical Settings

**DOI:** 10.2196/mhealth.2268

**Published:** 2013-04-23

**Authors:** Urs-Vito Albrecht, Marianne Behrends, Regina Schmeer, Herbert K Matthies, Ute von Jan

**Affiliations:** ^1^P.L. Reichertz Institute for Medical InformaticsHannover Medical SchoolHannoverGermany; ^2^Nursing DepartmentHannover Medical SchoolHannoverGermany

**Keywords:** medical informatics applications, nursing care, cultural deprivation

## Abstract

**Background:**

Communication between patients and medical staff can be challenging if both parties have different cultural and linguistic backgrounds. Specialized applications can potentially alleviate these problems and significantly contribute to an effective, improved care process when foreign language patients are involved.

**Objective:**

The objective for this paper was to discuss the experiences gained from a study carried out at the Hannover Medical School regarding the use of a mobile translation application in hospital wards. The conditions for successfully integrating these technologies in the care process are discussed.

**Methods:**

iPads with a preinstalled copy of an exemplary multilingual assistance tool (“xprompt”) designed for use in medical care were deployed on 10 wards. Over a period of 6 weeks, approximately 160 employees of the care staff had the opportunity to gather experiences with the devices while putting them to use during their work. Afterwards, the participants were asked to fill out an anonymous, paper-based questionnaire (17 questions) covering the usability of the iPads, translation apps in general, and the exemplary chosen application specifically. For questions requiring a rating, Likert scales were employed. The retained data were entered into an electronic survey system and exported to Microsoft Excel 2007 for further descriptive analysis.

**Results:**

Of 160 possible participants, 42 returned the questionnaire and 39 completed the questions concerning the chosen app. The demographic data acquired via the questionnaire (ie, age, professional experience, gender) corresponded to the values for the entire care staff at the Hannover Medical School. Most respondents (35/39, 90%) had no previous experience with an iPad. On a 7-point scale, the participants generally rated mobile translation tools as helpful for communicating with foreign language patients (36/39, 92%; median=5, IQR=2). They were less enthusiastic about xprompt’s practical use (36/39, median=4, IQR=2.5), although the app was perceived as easy-to-use (36/39, median=6, IQR=3) and there were no obvious problems with the usability of the device (36/39, median=6, IQR=2).

**Conclusions:**

The discrepancy between the expert ratings for xprompt (collected from the App Store and online) and the opinions of the study’s participants can probably be explained by the differing approaches of the two user groups. The experts had clear expectations, whereas, without a more thorough introduction, our study participants perceived using the app as too time consuming in relation to the expected benefit. The introduction of such tools in today’s busy care settings should therefore be more carefully planned to heighten acceptance of new tools. Still, the low return rate of the questionnaires only allows for speculations on the data, and further research is necessary.

**Trial Registration:**

This study was approved by the local institutional review board (IRB), Trial ID number: 1145-2011.

## Introduction

Patients with a different cultural background and language than their health nursing staff are more likely to be disadvantaged in their access to the health system [1]. These patients often have unfavorable health outcomes compared to those who can competently converse with their health care providers in the same language [2-5]. It can be speculated that the presence of a language barrier can result in increasing dissatisfaction from both the patients as well as medical staff, and may therefore negatively influence the quality of care. Several studies have shown evidence for these patients to have an increased risk of suffering from adverse events [5-8]. Also, problems caused by language barriers may unnecessarily increase costs, due to higher consumption of health care resources caused by misunderstandings [9,10] resulting in more frequent visits.

The various problems posed by not being able to overcome language barriers are often also encountered at the Hannover Medical School, which is a maximum care facility and, especially for certain specialties, often attracts patients from abroad. Although translators are usually called upon for communicating about important aspects of the treatment, particularly for uncommon languages not spoken by any staff members, they are not always readily available for everyday communication, which may unnecessarily complicate a patient’s care. Thus, one would expect that the staff would welcome any tool that can aid them when communicating with foreign language patients.

In this context, a mobile electronic translation tool that is ubiquitously available, hassle-free, and provides quick translations for users seems attractive at first glance. Several mobile device applications are already available for mobile devices (eg, MediBabble Translator [11] by NiteFloat, Inc, or Universal Doctor Speaker [12] by Universal Projects and Tools SL).

Nevertheless, little is known about the acceptance by the nursing staff or usability and efficiency of mobile translation tools in clinical settings and whether it is possible to properly integrate such applications into existing workflows. In order to be able to make future deployment of mobile devices and their preinstalled applications a success, it is important to identify the steps that need to be taken during the introductory phase in order to be able to address the needs, expectations, and fears of potential users (eg, benefits that users can expect, or limitations that might be encountered). We conducted a preliminary study at the Hannover Medical School to gain insight on the actual value of such apps in a real world setting based on staff feedback after using iPads equipped with an exemplary translation app.

## Methods

### Overview

For our study, 10 clinical wards selected by the nursing management were provided with iPads (one per ward) containing a copy of the “xprompt—multilingual assistance” application, as well as other reference material and applications. Since our study regarding the use of xprompt (descriptive, cross-sectional, post test only design) was part of a larger study dealing with the use of mobile devices such as iPads in nursing, the selected wards covered a wide range of surgical as well as non-surgical specialties, such as neurology and neurosurgery, urology, nephrology, plastic surgery, otolaryngology, pneumology, trauma surgery, thoracic surgery, and maxillofacial surgery. Both normal as well as private wards were included. After obtaining the informed consent from the staff, the iPads were distributed to the wards and the potential users were encouraged to install additional applications from the App Store or to research content on the Web.

Altogether, over a period of 6 weeks, about 160 staff members were given the opportunity to familiarize themselves with the device and its content, including xprompt. As the focus of the overall study was on the nursing staff, patients were not given the chance to use xprompt on their own on the provided iPads. The purpose of xprompt was simply to provide additional means for alleviating communication problems between the nursing staff and non-German speaking patients. After the trial phase, an additional, anonymous evaluation was conducted over the course of 2 weeks. The study was approved by the Institutional Review Board of the Hannover Medical School, Trial ID number: 1145-2011.

### The Application

“xprompt—multilingual assistance” is a commercial application (Blue Owl Software LLC, Boston, MA, USA) available for the iDevice ecosystem (ie, iPhones, iPads, and iPods). According to the developers, it was designed with the goal of improving the communication process between the nursing staff and patients in situations where language barriers exist [13].

The app was included into the study’s application portfolio based on the highly positive reviews it received from health care professionals. For example, the special review portal, iMedicalApps, stated that this app had “tremendous clinical utility to facilitate an interactive dialogue and maximize the healthcare provider-patient relationship” [14].

The application can be helpful in many different settings as it contains a large phrase set (800 phrases, currently available in 23 languages), covering nursing care as well as daily life communications. Although no official study on the quality of the content or the usability of the xprompt application is currently available, the available phrase set was deemed trustable since all translators had a medical education, and the quality of their translations had been verified by native speakers with a medical background [15]. Both the customer reviews given in the App Store and the positive ratings from iMedicalApps were interpreted as expert opinions.

**Figure 1:**
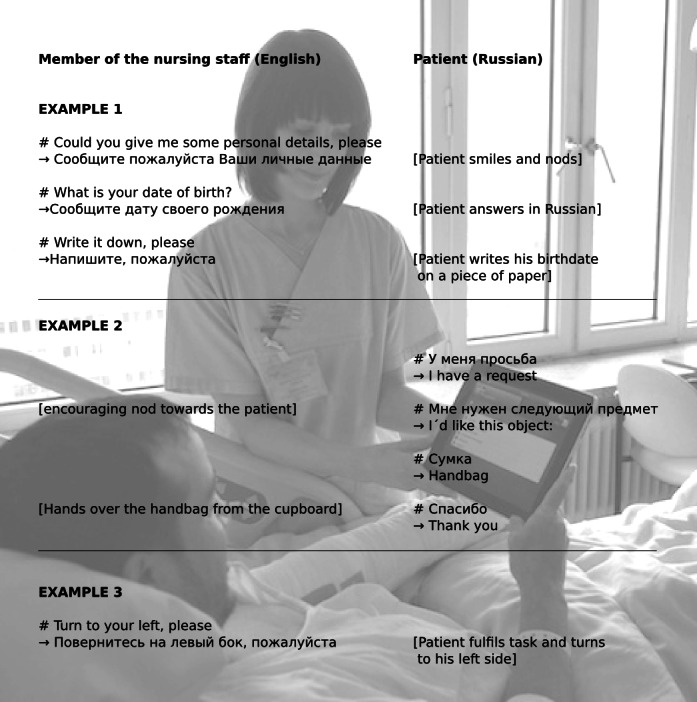
Three examples for basic communication between nurses (left) and patients (right) based on the phrases integrated into xprompt. “#” indicates the phrase chosen in the application, and “→” the corresponding translation. Notes in “[ ]” describe the reactions of the participants.

**Figure 2:**
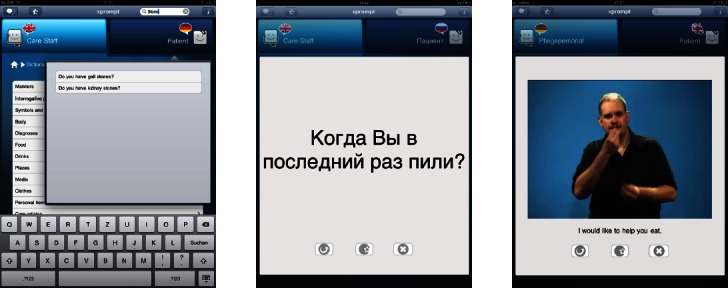
A search for specific phrases in xprompt (left), a phrase in Russian language (center), and video translation into British Sign Language (right).

**Figure 3:**
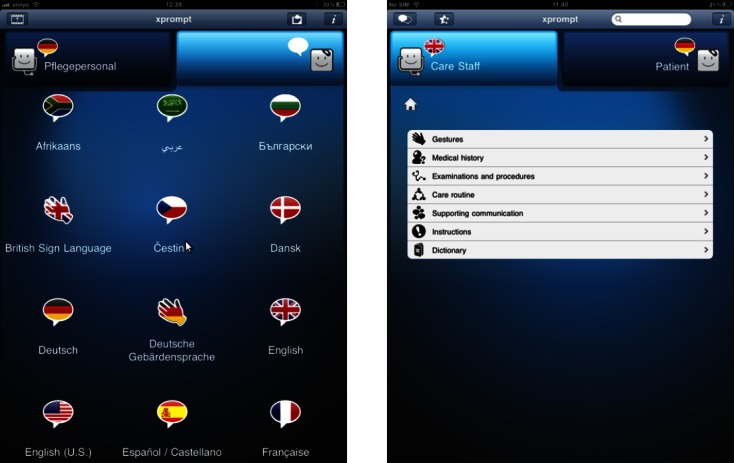
Choosing the languages to be used in xprompt (left). Typical menu entries for the care staff (right).

The application usage is simple. The phrases are provided in tailored menus for the nursing staff and the patients, grouped according to the situation in which they might be used with a quick navigation to the desired content. Via simple point and touch actions, selected phrases are translated into the target language. As an example, 3 short interactions are shown in Figure 1. The results are presented in text form as well as either an audio output for spoken languages or video sequences for sign languages (Figure 2). Users have the option to respond by using the language of the person they are interacting with by changing the language mode and choosing the desired phrases. Source and target languages can be freely combined (Figure 3).

### The Questionnaire

After the trial phase of the study, the aforementioned evaluation was performed. In total, the questionnaire contained 17 questions relating to either xprompt or basic usage of the devices. The questions we asked about using xprompt were integrated into the survey for the larger study that covered a wide range of aspects dealing with the general usage of mobile devices in a clinical setting. Due to time constraints, it was not feasible to cover all separate aspects with individual standardized usability questionnaires, which would have been desirable to guarantee comparability with other studies. We did not want to overly tax the patience of the personnel who agreed to answer our survey.

The Likert scale was employed for rating the intensity of various question items (eg, “not at all”, “very little”, “a little”, “somewhat”, “fairly”, “strongly”, “very strongly” for a 7-point scale, and “never”, “rarely”, “sometimes”, “often”, “very often” for a 5-point scale). Also, non-verbal 6- or 7-point Likert scales were used to discriminate between poles (eg, “several times per day” and “never”, or “totally agree” and “totally disagree”). The topics covered the iPad usage within the project as well usage of the iPad in general, the availability of the distributed devices during the project, the experienced usability of the device, relevance of the iPad related to work, expectations of working with the iPad in the future, and the general attitude towards the usefulness of translation apps (7 items). Another group of two items dealt with the usability aspects of xprompt. A third group asked about the experience of the usage of xprompt in communication with patients and colleagues and in which way the application was helpful for overcoming communication problems between the nursing staff and patients (3 items). Regarding sociodemographic information, the study participants were asked about their age in years, their gender, work experience in years, and whether they had had any previous experience with the iPad before the study (4 items). The participants were also encouraged to provide short free-text comments to express their overall opinion on xprompt (1 item). The questionnaire was pretested using the thinking-aloud technique with 4 members of the nursing staff. Filling out the interview took no longer than 10 minutes time in the pretest.

Due to the anonymous nature of the evaluation, it was impossible to initiate individual follow-up attempts. Anonymization was granted by providing a randomized digital alpha-numerical code written on the questionnaires. After filling out the interviews, the participants returned them using unmarked envelopes that were collected on each ward. All envelopes were returned to the Institute for Medical Informatics where they were opened and the documents were scanned for automatic data collection using an electronic survey system [16]. The data were stored anonymously on a password-protected computer with no connection to the Internet. The survey and the evaluation software were also password protected. Subsequently, the data were exported to Microsoft Excel 2007 for further descriptive data analysis.

The available free text comments were analyzed systematically with respect to the actual use of the system, the type and content of any communication/interaction that had taken place using xprompt, the type of patients, problems that occurred, and whether there were any reasons for not using the system in specific situations. Additionally, to gain deeper insights into how the nurses had actually used the system, we asked 5 members of the nursing staff to reveal themselves as participants, regardless of whether they had employed the system for their daily work or not. These 5 users were also asked to fill out the system usability scale (SUS) questionnaire by Brooke [17], a standard tool for usability testing following ISO 9241/11, which can be used to determine effectiveness, efficiency, and user satisfaction of a system [18]. Although the SUS questionnaire contains only 10 questions (Table 1) and leads to a single score, according to [19], it actually contains 2 factors that measure usability and learnability aspects, which can also be used separately with similar effectiveness. The SUS score correlate well with the individual scores of usability and learnability, which are also intercorrelated. In [20], this 2-dimensional structure of the SUS was confirmed. When answering the questionnaire, users could choose the desired value on a 5 point Likert scale that ranges from “totally disagree” to “totally agree”. Based on the answers, a simple score (range: 1-100) was calculated that could be used to rate the user-friendliness of the system, with 100 representing the ideal value [17]. The SUS questionnaire was translated into German language and used in this study.

**Table 1:** Median and IQR for all SUS items (original scores), based on N=5 interviews.

No.	SUS-Item	Median	IQR
1	I think that I would like to use this system frequently	3	1
2	I found the system unnecessarily complex	2	1
3	I thought the system was easy to use	4	1
4	I think that I would need the support of a technical person to be able to use this system	2	1
5	I found the various functions in this system were well integrated	3	1
6	I thought there was too much inconsistency in this system	3	1
7	I would imagine that most people would learn to use this system very quickly	4	2
8	I found the system very cumbersome to use	2	2
9	I felt very confident using the system	4	1
10	I needed to learn a lot of things before I could get going with this system	2	0

## Results

From all potential users who were given the opportunity to gather firsthand experiences with the devices and their software, 42 participated in the final evaluation. Of these, 39 also answered the questions regarding xprompt. It was not possible to determine the exact number of employees who had the chance to work with the iPads since there were no data about vacations and sick leave. Still, it can be assumed that the number of employees amounts to approximately 160. Thus, the return rate of completely filled out questionnaires was approximately 24% (39/160). For each question, only the participants who provided a valid answer were counted.

Even with the low return rate, the study population was a typical sample of the nursing staff at Hannover Medical School, based on the proportion of female employees compared to a previous statistic. At the end of 2012, 83% of the total 2596 employees were female, which parallels with the demographics of this study, where 87% (34/39) of the participants were female. Participants also had a comparable age distribution of 69% (27/39) up to 45 years of age and 31% (12/39) for older participants (Table 2). Other important demographics were 50% (19/39) of the participants had a work experience of 10 years or less. 90% (35/39) of the participants were regular nurses and 10% (4/39) were specialized nurses (Table 3). 90% (35/39) of the interviewed nursing staff stated that they had had no experience with the iPad before the project.

One out of four nurses (9/38) worked part time and thus did not have as much of a chance to work with the provided iPad (Table 3). Since nursing education in Germany does not take place at a university level, 74% (28/38) of those answering the survey had reached an education equivalent of Secondary School Level I before their training in nursing (Table 3).

**Figure 4:**
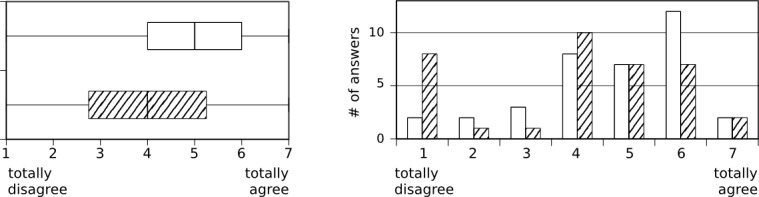
Details regarding usefulness. White bars: a translation system like xprompt is very helpful for communication with non-German speaking patients. Cross-hatched bars: with xprompt, I was able to provide assistance with language problems.

**Figure 5:**
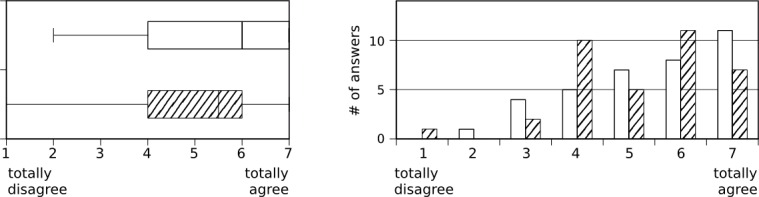
Ability to use and learn xprompt. White bars: the handling of xprompt is uncomplicated. Cross-hatched bars: I was able to learn the usage of xprompt in a short time.

**Figure 6:**
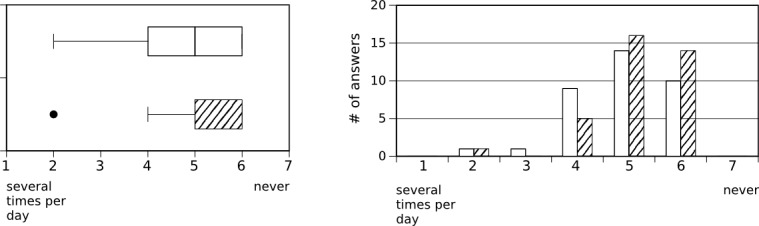
Frequency of using xprompt with patients (36 answers, cross-hatched bars) and alone or with colleagues (35 answers, white bars).

The results from the interviewed group showed that 76% (27/36) had “rarely or never” used xprompt during the study, and 71% (27/38) stated that the iPad was almost always available when desired. Mobile translation tools such as xprompt were found to be helpful for daily communication with foreign-language patients (median=5, interquartile range (IQR)=2, scale 1-7, N=36) although xprompt itself received more neutral ratings (median=4, IQR=2.5, scale 1-7; N=36, see Figure 4). This difference cannot be attributed to the usability aspects of the application (see Figure 5) as xprompt was perceived to be easy-to-use (median=5, IQR=2, scale 1-7, N=36) and users did not have to spend much time to familiarize themselves with the application (median=5.5, IQR=2, scale 1-7, N=36). It was primarily used with patients (median=5, IQR=1, scale 1-6, N=36), but also with colleagues (median=5, IQR=2, scale 1-6; N=35, see Figure 6).

The device usability was rated positively by 90% (32/36, median=6, IQR=2) and 33% (12/36) assumed the iPad as relevant for their daily work routine while 19% gave it a more neutral rating (7/36, median=4, IQR=3). Altogether 82% (27/33, median=4, IQR=1) stated they would be able to use the device. Of these, (67%, 22/27) required no further introduction, while 15% (5/27) felt that they required the manual for continued use at their wards.

Nevertheless, Hannover Medical School—a facility providing maximum care—attracts patients from abroad. According to internal statistics, there are between 300 and 400 foreign patients per year from a variety of countries whose treatments are not being paid for by the German health insurance system; these are usually interested in certain specializations that are not available where they come from or in being treated by specific experts. Patients with a migration background who live in Germany are often able to communicate sufficiently. Thus, patients requiring help with communication are not evenly distributed between all wards and as was to be expected, during our evaluation, some of the wards that had been equipped with iPads simply did not have any patients that were unable to communicate due to inadequate language skills. All in all, the patients fitting the profile were a heterogeneous group of both sexes and ages ranging from the early twenties to the late seventies. They were mainly treated on surgical but also gastroenterological wards and were of Libyan, Syrian, Turkish, Pakistani, Polish, Ukrainian, and Russian origin.

The analysis of the free text comments and additional in-depth interviews made it clear that xprompt was used for professional communication during patient care. The emphasis was on basic communication/interaction (examples given in Figure 1). There were also several attempts to communicate about more complex issues, although when attempting to provide information about delicate procedures (eg, surgical procedures or other interventions), the members of the nursing staff often indicated that they had avoided using the system.

Some elderly patients had problems to use and to “get in touch” with the devices since they were unfamiliar with such technology. Also, older members of the nursing staff were more cautious and skeptical about using the devices and xprompt. In some cases, only the nursing staff was able to really use the app since the patients were unable to read the menus due to either visual impairment or analphabetism. In these cases, since there is always audio output available, it was at least possible for the staff to provide patients with some basic information if they were not hard of hearing as well. At times, the desired target language was not available. The staff also experienced problems when trying to explain the menus to foreign language patients. In one case, a patient who had been severely traumatized by war refused any communication and in another case, poor overall compliance did not allow any conclusions to be drawn about whether communication worked at all.

On some wards, another reason for not employing xprompt was that a single iPad per ward was simply not sufficient; the staff would have liked to have separate iPads for each nurse since it was a hassle to share a single device and to have to ask around for the device whenever it was needed.

Also, some nurses stated that their problems with using xprompt and the iPads stemmed from being unfamiliar with such technology and that they had not received any introduction to the devices and the installed software; colleagues , mostly head nurses, who had been trained, were often not available when they needed help. One suggestion given in the free text answers was to offer several training sessions open for all staff members, another request was to provide direct bedside teaching. Although an introductory booklet that covered the basics had been distributed along with the devices, it was not always readily available. Other key statement were that at times, the tablet PCs had simply been locked away when someone wanted to use them or that they could not be used as frequently as desired due to the high workload, which the users found unfortunate.

**Table 2:** Male-to-female ratio and age distribution in the final survey (N=39) and for the Hannover Medical School (N=2569, data for 2012 obtained from the human resources department).

Category		Survey n (%)	Hannover Medical School n (%)
**Sex**			
	Female	34 (87)	2126 (83)
	Male	5 (13)	443 (17)
**Age**			
	Up to 45 years	27 (69)	1721 (67)
	46 years and older	12 (31)	848 (33)

**Table 3:** Job functions (N=39), work experience (N=38), work model (N=38), and educational level of the participants.

Category		n (%)
**Function**		
	Regular nurse	35 (90)
	Specialized nurse	4 (10)
	Trainee	0 (0)
**Work experience**		
	Up to 5 years	11 (29)
	6-10 years	8 (21)
	11-15 years	5 (13)
	16-20 years	1 (3)
	>20 years	17 (34)
**Work model**		
	Part time	9 (24)
	Full time	29 (76)
**Educational level**		
	Secondary school level I	28 (74)
	Secondary school level II	8 (21)
	College / applied sciences	2 (5)
	University	0 (0)

## Discussion

### Principal Findings and Conclusions

**Figure 7:**
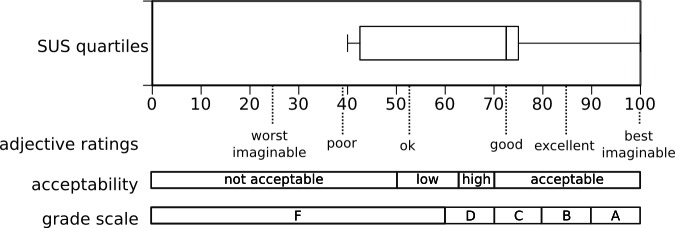
Score of the SUS sub-study [21]. The SUS score (median) was 72.5 (IQR=34.5). Three female and two male members of the nursing staff (N=5), 18-25 years of age (2/5), 26-35 years of age (1/5), 36-45 years of age (1/5), and 46-55 years of age (1/5) voluntarily answered the SUS questionnaire. All were regular nurses working in a full time scheme. The work experience included time spans of under 5 years (2/5), 11-15 years (1/5), and 26-30 years (2/5). Educational levels were Secondary School Level I (2/5) and II (3/5).

The results showed an obvious discrepancy between the expert assessments of xprompt, stated in the user comments on the App Store [13] and on iMedicalApps [14] and the actual usefulness attributed to xprompt by the participants of our study. When looking at the other ratings given for xprompt, it can be ruled out that individual problems with the usage of xprompt were the reason for the neutral ratings it received with respect to supporting the communication process. Users at Hannover Medical School perceived the translation app as useful for basic communication; in this context, xprompt’s restriction to basic, predefined phrases was accepted, that is, one participant stated “small things could be sorted out but for complex issues, due to time constraints caused by high workload, it seems sensible to wait for an interpreter”. Another statement given in the free text answers was that the application was well structured and the menu could easily be used for navigating the content. One user thought that over time, he would become familiar with the application and thus be more at ease and feel more secure in using it. Since a considerably large proportion of participants reported that they did not use the system at all, we conducted additional short-scaled usability testing based on the SUS specified by Brooke [17] in order to determine whether these findings lead to any distortions concerning the usability, task orientation, and general user satisfaction. The results of the SUS evaluation (SUS score=72.5) showed that xprompt seems well adapted for its task (Figure 7). Based on the answers of 5 volunteers who participated (Table 1), the suspected distortion could not necessarily be confirmed and it can be assumed that it does indeed not play an important role. According to Nielsen and Virzi, even small sample sizes are sufficient for detecting major usability problems [22,23]. Nevertheless, since xprompt is not overly complex, there should be no major deviations from results one might obtain using a larger number of participants; other reasons aside from usability seem to be responsible.

As shown by previous studies, in health communication situations where a language barrier existed, professional translators were not always requested even if they were available [24-26]. Diamond et al hypothesized that the staff make “decisions about interpreter use by weighing the perceived value of communication in clinical decision-making against their own time constraints” [26]. Results from our study supported this claim, where the great time pressure that medical staff are subjected to was reflected in their assessments of xprompt. Evidence for this can be found in the free text entries of the interviewees. For example, one female participant specified that “it is simply not possible to spend ‘hours’ on language problems because shortness of time”.

Adequately dealing with language problems was often viewed as too time consuming, but the nursing staff generally viewed translation tools such as xprompt as useful for solving such problems and saving time. But initially, using such tools always requires additional work to adapt to the change. During the study, it was obvious that the nursing staff had difficulties in explaining the program’s use to foreign language patients, as stated by one of the participants, “without any explanation, the application was too complicated to work with”. Although a language-independent video tutorial explaining its use was integrated in the application, the nurses did not use it at the bedside due to their perception of time constraints and too much personal distance to the patient when just passively showing a video instead of interactively introducing the application. To properly show the application’s functionality, the nursing staff would not only have to understand the app but to plan how to best present it to the patients and spend additional time on the actual explanation.

The above appears to indicate that ultimately, a distinction must be made between the individual use of xprompt and its use in the context of nursing care. For integrating it in the daily routine of inpatient care, detailed instructions with respect to the application’s use must be provided.

It can be assumed that individual users who installed xprompt on their own initiative had clear expectations about the program. They were searching the App Store for solutions to a specific problem they had encountered, that is, an “always available mobile medical translation”. With proper research on the topic, they were able to learn about and compare strengths and weaknesses of the available solutions. When downloading the application and testing it, there would not be excessive expectations regarding the functionality of the app, biasing their opinion about its potential. Users would have a realistic idea of the application’s features and limitations they might encounter.

Due to their research into possible solutions, the individuals who had voluntarily chosen to use xprompt had a “head start” and were well informed about what they could expect from the application. The users in our study setting did not actively choose to use xprompt and did not have as much information about the app or similar competing products. Rather, the nursing staff was provided with a solution right away, without being able to familiarize themselves with all the aspects (ie, possibilities and limitations) of applications for overcoming the language barrier. Instead they found themselves in a situation with very little time but much work dealing with the patients, while having to personally understand the usage and functionality of the application and at the same time already having to explain it to the patients.

The results regarding the deployment of xprompt showed that, when introducing new technologies, it is especially important to adequately train the nursing staff and adapt the training according to their job requirements. Appropriate steps must be taken to ensure that users do not simply consider the provided technology as a gadget rather than as a useful tool. They must become aware of the opportunities mobile technologies can offer. Otherwise there will be little chance to integrate such tools in their daily routine. Thus, simply training users and providing information about how to use the devices and the apps installed on them can contribute to an improved acceptance. One approach that can be taken to establish improved acceptance on new technologies is to provide realistic information about the capabilities of specific applications and technologies, such that users will not overestimate the power of the technology and be appreciative of the advances brought by the technology.

The use of mobile translation tools may certainly support the communication between patients and nursing staff in the absence of a common language. Nevertheless, certain restrictions must also be observed, for example, the application may not be used in highly sensitive situations, especially if a patient’s life is in danger. The main objective was to alleviate health communication problems posed by language barriers and thus promote empowerment and medical autonomy of the patients in situations where no interpreter can be reached.

### Limitations and Further Research

Due to the limited nature of the study with only one iPad per ward, patients did not have much opportunity to familiarize themselves with the application. As the application was used with patients of many different cultural and linguistic backgrounds and the focus of the study was on the acceptance of the application by the nursing staff, we refrained from preparing separate questionnaires for patients for each of the available languages.

Although one might argue that the low number of responders in the survey is correlated to poor interest toward the application, it is more likely due to the way the iPads and their software were introduced on the wards. At the end of the project “iPads in Nursing” in which our evaluation of xprompt was embedded, the iPads were collected from the wards with the aim of using them in other projects. Since then, there have been numerous urgent requests by wards that traditionally have a large number of foreign speaking patients, such as the department of trauma surgery, to again provide them with iPads equipped with xprompt and we happily complied whenever possible. When asked what makes a solution such as xprompt attractive, the requesting personnel stated for example that they needed it to communicate with Arabic or Russian speaking patients, and that due to the program’s clear structure, it really simplified the basic communication process if no other help was available. In order to provide deeper insights, we would like to conduct a more detailed study using a pre/post design, specifically addressing the limiting factors we identified. Such a follow-up study might include the introduction of solutions to the issues discussed above, including time constraints and the education of nurses regarding mobile technologies, in order to influence the perceived usability. Also, this time, the focus should be only on those wards that traditionally treat a large number of foreign patients.

Of course, staff and patients have different motivations when using xprompt. For patients, their expectations, needs, and fears during a medical emergency, a diagnostic procedure, treatment, or care situation is very different from what their professional counterparts perceive. Based on their professional experience, nurses will often already have found some other, possibly nonverbal, way to ensure basic communication. Therefore, in our forthcoming, more extensive study, we would also like to put an additional emphasis on aspects relating to patients using systems such as xprompt.

## Abbreviations

IQR: interquartile range
SUS: system usability scale
