# Metadata Correction: Usage of Multilingual Mobile Translation Applications in Clinical Settings

**DOI:** 10.2196/mhealth.2866

**Published:** 2013-08-07

**Authors:** Urs-Vito Albrecht, Marianne Behrends, Regina Schmeer, Herbert K Matthies, Ute von Jan

**Affiliations:** ^1^P.L. Reichertz Institute for Medical InformaticsHannover Medical SchoolHannoverGermany; ^2^Nursing DepartmentHannover Medical SchoolHannoverGermany

The authors of "Usage of Multilingual Mobile Translation Applications in Clinical Settings" (JMIR Mhealth Uhealth 2013 (Apr 23); 1(1):e4) inadvertently omitted Regina Schmeer (Nursing Department, Hannover Medical School) from the list of authors during the submission process. The author Schmeer should have been added after Marianne Behrends in the originally published manuscript. The footnote "all authors contributed equally" extends to the omitted author. The omitted author certified that there are no conflicts of interests to add. This error has been corrected in the online version of the paper on the JMIR website on August 7, 2013, together with publishing this correction notice. This was done before submission to PubMed or Pubmed Central and other full-text repositories.

